# Cases of Neuralgia

**Published:** 1823-06

**Authors:** Benjamin Hutchinson


Art. V.—*Cases of Neuralgia.* Communicated in a Letter to Dr. Macleod, by Benjamin Hutchinson, Esq.

I have been favoured with the history of the following case of neuralgia by Charles Wright, Esq. of Wolverhampton, and shall take the liberty of detailing it in his own language.

"For the last six or seven years, I have been occasionally subject to a violent complaint in my head. The exact seat of the disease is the right temple, and the pain comes on periodically.

"About nine or ten o'clock in the morning, a most violent beating or throbbing begins in my temple, strikes directly through my right eye to nearly the centre of my forehead, and continues without intermission until about five o'clock in the evening, when it leaves me.

"I have been frequently confined to my room a month or five weeks, during great part of which time I could not bear the light, nor suffer any person to make the least noise: indeed, when the paroxysm has been most severe, I could not bear any one to speak to me.

"Bleeding, blistering, bathing with hot water, and innumerable experiments, I have tried with little success; but relief has generally been obtained when I have been so reduced by pain that I have been unable, to all appearance, to bear more. When I have been sufficiently recovered to use exercise, I have always found my head so swollen as to be unable to wear my usual hat for a considerable length of time.

"In October, 1820, I heard of a pamphlet published by Mr. Hutchinson, of Southwell, which I immediately purchased. I was then in tolerably good health, but very soon afterwards I felt symptoms of my old complaint, and dreading (as I always do) an attack, I immediately began to take the carbonate of iron; but, as I only took half a drachm twice a day, I found no benefit: indeed, the disorder appeared to gain ground for two or three days. I then took a drachm twice a-day for more than a week, when the pain quite left me. If I go out in foggy weather, or in a very sharp wind, the old symptoms will return in a trifling degree; and I have immediate recourse to one drachm of the carbonate of iron twice a-day, with the full and most happy effect of preventing an attack of any violence. At one period of this horrid disease, I was fearful of losing the sight of my right eye, and my head sometimes shook like a person who had the palsy. Whether I must attribute it to the iron, I cannot say, but my health has certainly been much better since I took it. I am, &c.

(Signed) "CHARLES WRIGHT."

The two following cases have recently occurred in my own practice:

Miss Tongue, residing at Halloughton, in the county of Nottingham, the subject of the following brief narrative, a young lady between twenty and thirty years of age, began to experience intermitting, and not very violent, pains in the right side of her face, at so remote a period as six or seven years ago, which, being considered by herself and her friends of a rheumatic character, or probably rather the effect of failing teeth, were not deemed of sufficient importance to excite any marked attention. The very long continuance, and the dreadfully increasing severity of this pain, at length compelled this young lady to seek for some relief; and, induced by the circumstance of two young ladies, friends of my patient, afflicted with the same malady, and cured by the methods which I had publicly recommended, I was consulted on the case in the middle of the month of December, 1822. My patient was of a pallid complexion; of her general state of constitution not complaining; the catamenia and bowels observing a regular and very healthful action. She described her pains as coming on in an instant; sometimes above, and at others immediately below, her right eye; sometimes at the side of her nose; at others extending over her upper lip, producing swelling and inflammation of her gums, and considerable discoloration of her face. The pains were described as of the most lancinating and shooting kind, frequently depriving her of rest, and of the power of mastication. She had the indulgence and luxury of but few intervals of ease, and was in the greatest despair of finding a remedy for her sufferings. I could have no hesitation in pronouncing this a genuine and most severe example of tic douloureux. The immediate seat of the nerves thus morbidly affected, and the most probable mode of affording relief to their agonies, were equally manifest. The superior maxillary, as it passes through the foramen rotundum, the first branch of the fifth pair, the ophthalmic, and that branch of the ophthalmic which goes to the lachrymal gland, were the nerves principally instrumental in the production of these miseries. The third branch of the fifth pair, the lower maxillary nerve, and the portio dura of the seventh pair, distributing its ramifications to most parts of the face, and communicating with several of the fifth pair, were partakers also in the torments of this young lady. Every mode which had been adopted having failed to give any relief, I began my curative assault by a brisk operation on the primæ viæ, by means of an evening dose of calomel, succeeded by a saline purge; after two doses of which, I prescribed one drachm of the subcarbonate of iron three times a-day, and two anodyne pills at bed-time, consisting principally of the extracts of hyoscyamus and stramonium. After a few days' perseverance in this plan, a very considerable diminution of pain, both in its duration and severity, was most happily experienced, without producing the least unpleasant effects or inconvenience of any kind. This state of convalescence was daily and most satisfactorily improving, until my patient was unfortunately exposed to the rigorous cold of one of the very winterly days of last January, from which she experienced a smart catarrhal attack, and a recurrence of the pain in her face. A few days' continuance of febrile symptoms rendered the use of the iron improper, but which was resumed with confidence on their removal. My patient again was rendered happy by the gradual abatement of her formerly inveterate enemy, and in the course of another fortnight was perfectly well. I have had the pleasure of seeing the young lady within the last day or two; and she has the satisfaction of assuring me, that, on exposure to the late very inclement weather, she has felt no return of her pains.

Miss Mary Clarke, of Normanton, near Southwell, twenty-two years of age, of a fair and florid complexion, has been occasionally subject to very severe painful affections, during the last six or seven years, in the left side of her face. My opinion and advice being requested, I accurately examined the state of the mouth and teeth, and found one of the lower molares in a very diseased state; but, as the pain had never assailed that particular tooth, and was generally situated in the upper jaw, I could do no otherwise than acquit it as being the cause of my patient's very distressing sufferings. The general state of her health was unimpaired, and her painful paroxysms were always exasperated by the irritation of cold air, or the application of cold water either to her mouth or face. Her nights were usually restless; and, at one or two o'clock every morning, she was awoke by the most tormenting spasms attacking the parts in the immediate neighbourhood of the ear, the side of the nose, the under and upper parts of the orbit of the eye, and sometimes extending its influence to the integuments of the parietal bone of that side. The pain usually began with a sensation of twitching and convulsive agitation, observing a regular ticking, similar to the oscillations of a clock pendulum. These tortures would continue one, two, or more hours; were never uniform in the term of their duration, nor in the severity of the pain, but invariably exact in their periodical visitations. In this state of misery my young patient continued for five or six years; her days being frequently rendered most irksome, as well as her nights most distressing, and all the usual routine of medicine and anodyne applications proving equally inert. The tortures of this young lady were manifestly situated in the second branch of the fifth pair of nerves; in the portio dura of the seventh pair, denominated of late, by Mr. Charles Bell, the respiratory nerve of the face; those branches of the fifth pair distributed among the bones of the face to the eyes, nose, mouth, and tongue; the ophthalmic branch; the superior maxillary; the posterior auris; the stylo-hyoideus; and that branch which supplies some of the deep muscles, and joins the laryngeal branch of the eighth pair. The functions of the chylopoietic and of the uterine system were very healthily performed; and I commenced my attack on this painful and truly distressing case by a mild dose of calomel, succeeded on the following morning by an aperient draught, which operated satisfactorily. I then prescribed one drachm of the ferri subcarbonas to be taken three times a-day, mixed in honey or treacle; and an anodyne at night, consisting of the extracts of stramonium, hyoscyamus, conium, and humulus, with a small proportion of James's powder. On the second night after the adoption of this plan, my patient's pains had very considerably abated; and, by a regular perseverance during the ensuing six weeks, a progressive state of amendment took place, until the disease became wholly subdued; and she is now enjoying the blessing of a perfect liberation from all her former neuralgic tortures.

Captain PARKER, of the Royal Artillery, Woolwich, has communicated a history of his own case, in the following letter:

"*Woolwich; 14th April,* 1823.

"Dear Sir,—I beg to acknowledge the receipt of your letter, requesting a statement of my case of tic douloureux, which I feel great pleasure in transmitting you.

"I am now between thirty-three and thirty-four years of age. As early as twelve, I was afflicted occasionally with an intense pain in and over my right eye, as if the eye had been torn out, and the socket filled with boiling lead. This pain always came on and left me daily, during the attack, at particular hours, with the most astonishing regularity. Emetics used, in those early days of my complaint, to give me relief. As I got older, the pains ceased to observe this regular intermission, and extended to my nose and face; and for the last eighteen years I have more or less suffered the greatest torments, though I was at one time two years without any very serious attack. Within the last three years, however, my sufferings have been constant and dreadful: I have tried, in very large doses, the bark, arsenic, and (I think) the subcarbonate of soda; plasters of all sorts, leeches, blisters, and particularly a plaster made by a nun in Paris, (which acted like a charm upon Mr. Fellowes, one of the greatest sufferers from tic douloureux in England;) all without the slightest effect. About two years ago I was strongly advised by Mr. Beard, at that time a surgeon in the Artillery, to make a trial of the medicine which you have so happily recommended,—the carbonate of iron. Lord Carlisle, also, in a letter to an aunt of mine, advised me to try it, and to read your pamphlet. I accordingly did so, and certainly with much success; but such was the obstinate prepossession in my mind that nothing could effectually relieve me, that I never gave it a fair trial, leaving it off too soon, and probably not observing sufficient regularity in my habits of living during the time that I was taking the medicine.

"In the beginning of last January, in consequence of sleeping in a damp bed, I was taken very ill with a severe rheumatic fever, which brought on a most dreadful attack of my old enemy,—in short, so bad, that I was at times rendered completely distracted. Sir Henry Halford, who attended me, so soon as the fever was sufficiently subdued, immediately gave me the carbonate of iron,—I think, to the quantity of eight scruples a-day, in three doses. I may not, probably, be quite correct in the quantity; but I sometimes took even more than Sir Henry desired me. From that period to the present I have never had a relapse, and, from certain feelings I experience, but which I am unable to describe, I am convinced that I am cured. I intend, however, occasionally to take six scruples of the iron a-day for a fortnight, even should I continue well. I left it off after taking it three months. I regret, from my never having kept any memoranda, that my statement is so general; but I shall at all times be most happy in answering any questions you may wish. I remain, &c.

(Signed) "J. PARKER."

"P. S.—I ought, perhaps, to mention that, when I was about nine years of age, my nose was broken by a blow from a cricket-bat."

